# Immobilization of Polyiodide Redox Species in Porous Carbon for Battery-Like Electrodes in Eco-Friendly Hybrid Electrochemical Capacitors

**DOI:** 10.3390/nano9101413

**Published:** 2019-10-03

**Authors:** Qamar Abbas, Harald Fitzek, Hartmuth Schröttner, Sonia Dsoke, Bernhard Gollas

**Affiliations:** 1 Institute for Chemistry and Technology of Materials, Graz University of Technology, 8010 Graz, Austria; bernhard.gollas@tugraz.at; 2 Graz Centre for Electron Microscopy, Steyrergasse 17, 8010 Graz, Austria; harald.fitzek@felmi-zfe.at (H.F.); hartmuth.schroettner@felmi-zfe.at (H.S.); 3 Institute of Electron Microscopy and Nanoanalysis, NAWI Graz, Graz University of Technology, Steyrergasse 17, 8010 Graz, Austria; 4 Institute for Applied Materials (IAM), Karlsruhe Institute of Technology (KIT), Hermann-von-Helmholtz-Platz 1, 76344 Eggenstein-Leopoldshafen, Germany; sonia.dsoke@kit.edu

**Keywords:** hybrid electrochemical capacitor, redox active electrolyte, polyiodide immobilizaton, battery-like electrode, charge transfer, activated carbon

## Abstract

Hybrid electrochemical capacitors have emerged as attractive energy storage option, which perfectly fill the gap between electric double-layer capacitors (EDLCs) and batteries, combining in one device the high power of the former and the high energy of the latter. We show that the charging characteristics of the positive carbon electrode are transformed to behave like a battery operating at nearly constant potential after it is polarized in aqueous iodide electrolyte (1 mol L^−1^ NaI). Thermogravimetric analysis of the positive carbon electrode confirms the decomposition of iodides trapped inside the carbon pores in a wide temperature range from 190 °C to 425 °C, while Raman spectra of the positive electrode show characteristic peaks of I_3_^−^ and I_5_^−^ at 110 and 160 cm^−1^, respectively. After entrapment of polyiodides in the carbon pores by polarization in 1 mol L^−1^ NaI, the positive electrode retains the battery-like behavior in another cell, where it is coupled with a carbon-based negative electrode in aqueous NaNO_3_ electrolyte without any redox species. This new cell (*the* *iodide-ion capacitor*) demonstrates the charging characteristics of a hybrid capacitor with capacitance values comparable to the one using 1 mol L^−1^ NaI. The constant capacitance profile of the new hybrid cell in aqueous NaNO_3_ for 5000 galvanostatic charge/discharge cycles at 0.5 A g^−1^ shows that iodide species are confined to the positive battery-like electrode exhibiting negligible potential decay during self-discharge tests, and their shuttling to the negative electrode is prevented in this system.

## 1. Introduction 

Electrochemical capacitors (ECs) can be charged and discharged thousands of times and are preferred energy storage and delivery devices in applications, where burst-mode power delivery is required [1,2,3,4], such as short route buses [5,6], stop/start (i-ELOOP) systems [7] and hybrid excavators [8]. In all these technologies, energy is recovered during deceleration and used when an object starts. However, the amount of energy delivered by the widely used electric double-layer capacitors (EDLCs) is low due to the physical storage of charges on both electrodes. According to the equation 1/C_cell_ = 1/C_+_ + 1/C_−_, the EDLC device capacitance is half of the capacitance of one electrode (C_cell_ = ½ C_+/−_). In contrast, hybrid electrochemical capacitors are the better choice, because one electrode works in a narrow potential range with a very high capacity and the other electrode in a wide potential window, consequently C_cell_ ≈ C_+/−_, exhibiting double the capacitance of their symmetric counterparts [9]. 

Owing to the low handling cost and good availability, aqueous solutions with near neutral pH are attractive electrolytes for ECs with potential windows of up to ca. 2.0 V [10,11,12,13]. A cell voltage larger than the thermodynamic potential window of water (*E*_O2/H2O_ = 1.23 V vs. SHE) is possible in aqueous electrolytes with neutral pH due to the negative carbon electrode exhibiting high over-potentials for the hydrogen evolution reaction [11]. It has been shown that also aqueous solutions containing redox active species can be used as electrolytes either by dissolving redox materials in water [14] or together with a supporting electrolyte of neutral pH, forming a so-called bi-functional electrolyte [15]. In cases where redox species are present in the electrolyte, the EC displays electrochemical signatures of a hybrid which is mainly controlled by the EDL electrode working in a large potential window with high capacity. For example, hybrid cells using hydroquinone-based redox-active electrolyte in H_2_SO_4_ have shown nearly twice the capacitance compared to a symmetric cell in H_2_SO_4_ [16]. Nevertheless, the hybrid cell with hydroquinone redox species displays a capacitance decay during cycling due to the shuttling effect leading to high self-discharge [17,18], which has been reduced to some extent by the use of ion-exchange membranes [19]. Similarly, hybrid capacitors with aqueous vanadyl sulfate (VOSO_4_) show high capacitance and a cell voltage of up to 1.4 V [20]. In order to simultaneously obtain a large potential window and high cell capacitance, redox species are mixed with a supporting aqueous electrolyte of near neutral pH [15,21,22,23,24]. The redox active species that work better in the presence of a supporting electrolyte are iodide salts mixed with aqueous lithium sulfate (Li_2_SO_4_) [15], aqueous choline chloride or choline nitrate-based supporting electrolyte [24]. The hybrid cells based on these bi-functional electrolytes exhibit nearly twice the capacitance compared to their symmetric counterparts and large potential windows of 1.5–1.6 V. 

Among the various hybrid ECs using redox-active aqueous electrolytes, the iodide-based one is probably the most suitable owing to the redox potential being very close to the equilibrium potential of the cell [25], which drives the battery-like positive electrode to work at a constant potential, while the negative electrode stores charge mainly in the electric double-layer (EDL). So far, hybrid ECs using either potassium iodide (KI) [14,26,27], Li_2_SO_4_ + KI [15], MnSO_4_ + KI [22] or choline nitrate + choline iodide-based electrolytes [24] have been proposed, and in all these systems, the potential of the positive electrode is nearly constant, while the negative electrode works in a large potential range using more than 90% of the cell potential window. Although the advantageous confinement of polyiodides inside the carbon pores of positive electrode prevents its self-discharge [28], the shuttling of iodine to the negative electrode cannot be fully prevented which affects the cycle-life of the hybrid cell [29]. In addition, a slight shift of equilibrium potential may drive one electrode to operate in the potential range of the other when discharging the cell down to 0 V and consequently deteriorate the cycle life, which must be controlled by appropriate mass balancing [30], in order to keep a constant capacity of each electrode throughout the cell operation.

The formation of polyiodides and their impregnation or entrapment inside the pore has been confirmed by Raman spectroscopy, when carbon-based materials are doped or impregnated with molecular iodine [31,32]. The interaction of iodine with the pi-electron cloud of carbon results in charge transfer forming a C-I complex [33]. Graphene has also been impregnated with iodine and the formation of polyiodide species (I_3_^−^ and I_5_^−^) has been confirmed as a result of charge transfer interaction of iodine with the graphene framework [34,35]. Charge transfer between carbon nanotubes and iodine has been confirmed with Raman spectroscopy and it has been suggested that iodine intercalates in carbon nanotubes [36,37]. The formation of polyiodides upon interaction of iodine with activated carbon has been confirmed by Amatucci et al., who used the C-I complex in hybrid capacitors [38]. Raman spectroscopy performed on carbon electrodes polarized in iodide-containing aqueous electrolyte confirmed the polyiodides confined within the nanopores. Besides, the upshift of D- and G-bands indicated a charge transfer between the carbon host and the iodide species [23]. From the foregoing, it is clear that activated carbon can not only be used for storage charge at the EDL just like other reported materials [39,40,41], but it can also be used to design battery-like electrodes, similar to some of the previously reported materials [42,43,44,45,46,47]. 

In this work, we take full profit of the iodine impregnated inside the nanoporous carbon electrode with a strong charge transfer forming C-I complex. Once the polyiodides are inside the pores, the carbon-iodide complex serves as a solid redox electrode, which has been used for designing a new hybrid capacitor in the neat aqueous NaNO_3_ electrolyte. By this way, the risk of iodine shuttling has been greatly reduced and parameters such as cyclability and self-discharge have been improved. For a positive electrode, carbon with a high surface area and a relatively large number of mesopores, which accommodates significant amounts of iodides, has been selected and a high surface area microporous carbon has been used as negative electrode to achieve high cell capacitance. 

## 2. Experimental 

### 2.1. Materials for Electrodes and Electrolytes 

The sodium iodide (NaI, 99.5%) and sodium nitrate (NaNO_3_, 99.8%) were purchased from Alfa-Aeser (Karlsruhe, Germany) and dried at 110 °C overnight before preparing the aqueous electrolytes of each salt at a concentration of 1 mol L^−1^ in de-ionized water. The pH of 1 mol L^−1^ NaI was 6.1 while it was 6.5 for 1 mol L^−1^ NaNO_3_ (both in the neutral pH range). Activated carbon cloth (ACC 507-20) from Kynol Europa GmbH (Hamburg, Germany) was used as a freestanding positive electrode while the negative electrode was prepared from a KOH activated carbon (named here KAC). The surface area and the pore size distribution of both carbons are given respectively in Table S1 and Figure S1 of the Supplementary Materials. ACC was used as the positive electrode owing to its more mesoporous volume which facilitated the accommodation of polyiodides, and the high micropore volume of KAC enabled the high EDL charge storage. In addition, the structural characteristics of pristine ACC by scanning electron microscopy (SEM) are given in Figure S2 which shows the presence of macropores on the surface of a fiber. To prepare the freestanding electrodes from KOH-activated carbon, 90 wt.% carbon powder was mixed with 10 wt.% carbon black (C65 from Imerys) as a conductivity additive and 10 wt.% of PTFE (60% dispersion in water from 3M^TM^, Dyneon^TM^) in ethanol. The mixture was continuously stirred at 60 °C using a magnetic stirrer with a hot plate until the solvent had completely evaporated and a dough was obtained, which was then pressed and rolled on a horizontal glass plate into a sheet electrode. Disc electrodes were punched out from the sheet and dried at 120 °C under vacuum in Büchi^®^ Glass Oven B-585 (Büchi Labortechnik AG, Flawil, Switzerland) resulting in a final thickness of 150 μm. For each hybrid cell assembled in this work, a positive to negative electrode mass ratio of 1:2 was used which allowed the cell equilibrium potential to remain at a near-constant value throughout the operation period. 

### 2.2. Assembling of Hybrid Cells and Physicochemical Investigation on Electrodes

The first hybrid cell named here as *hybrid cell 1* was assembled from an ACC positive electrode, a KOH-activated carbon-based negative electrode and 1 mol L^−1^ NaI as the electrolyte. After hybrid cell 1 was polarized up to 1.5 V by cyclic voltammetry at 2 mV s^−1^ and galvanostatic charge/discharge at 0.1 A g^−1^ (each technique repeated for 30 cycles to achieve an optimum conditioning of the electrodes), it was disassembled and the positive electrode was carefully transferred to another cell (further named in the text as hybrid cell 2). In hybrid cell 2, the electrochemically pre-polarized battery-like positive electrode was coupled with a KOH-activated carbon-based negative electrode similarly to hybrid cell 1, but with 1 mol L^−1^ NaNO_3_ as the electrolyte. Thermogravimetric analysis was performed on STA 449C Jupitar (Netzsch, Selb, Germany) under helium flow in the temperature range of 100 °C to 550 °C at a heating rate of 10 °C/min. To prepare the samples for TGA analysis, the polarized positive battery-like electrode was extracted from hybrid cell 1, cleaned with water, dried at 80 °C for 10 h and then hermetically sealed in aluminum crucibles. Samples of pristine carbon for TGA were prepared after drying at 80 °C for 10 h. Raman spectroscopy was performed on freshly polarized ACC positive battery-like electrodes extracted from hybrid cell 1. The battery-like positive electrode was transferred immediately after disassembling the hybrid cell to a Horiba Jobin Yvon LabRam 800 HR spectrometer equipped with a 1024 × 256 CCD (Peltier-cooled) and an Olympus BX41 microscope. All measurements were carried out using a laser wavelength of 633 nm. For comparison, Raman spectroscopy was also performed on a pristine ACC electrode. The carbon electrode’s surface was characterized by scanning electron microscope Zeiss Sigma 300 VP with a fully integrated EDS Detector Oxford SDD 80 possessing a special feature of large area mapping for detecting elemental distribution. 

### 2.3. Electrochemical Characterization of Hybrid Cells

Electrochemical investigations were performed in two-electrode cells with and without reference electrodes. Both were constructed from PTFE Swagelok-type vessels and 1.2-cm diameter current collectors made from nickel alloy C22, and 1.0-cm diameter disks as working and counter electrodes. A glassy microfiber separator (Whatman GF/A, 260 µm thick) soaked in 1 mol L^−1^ NaI or 1 mol L^−1^ NaNO_3_ electrolyte, which had been degassed for 15 min under reduced pressure at 24 °C, was sandwiched between the disc electrodes. A silver/silver chloride (Ag/AgCl in KCl_sat_, *E* = + 0.197 V vs. standard hydrogen electrode [SHE]) reference electrode was used to monitor the potential of the positive and negative electrodes. The potential range of the carbon electrodes in the two-electrode cells with reference were measured up to a cell voltage of *U* = 1.5 V by galvanostatic cycling with potential limits (GCPL) at 0.1 A g^−1^, 0.25 A g^−1^, 0.5 A g^−1^, and 1.0 A g^−1^ and by cyclic voltammetry with a sweep rate of *v* = 2 mV s^−1^. The hybrid cells in the two-electrode configuration were investigated by cyclic voltammetry (*v* = 2 mV s^−1^, 5 mV s^−1^, 10 mV s^−1^, 50 mV s^−1^) and GCPL (0.1 A g^−1^, 0.25 A g^−1^, 0.5 A g^−1^ and 1.0 A g^−1^) up to *U* = 1.5 V. A VMP3 multichannel potentiostat/galvanostat (Bio-Logic Instruments, Seyssinet-Pariset, France) was used for all electrochemical measurements. The gravimetric capacitance *C* was calculated by integrating the surface area under the galvanostatic discharge curve [48,49] and expressed per total active mass of the two electrodes (F g^−1^). The energy efficiency was estimated by the ratio of the integrated surface area under the galvanostatic discharge and charge curves. The long-term performance was also evaluated by estimating the capacitance evolution during 5000 galvanostatic (0.5 A g^−1^) charge/discharge cycles up to a cell voltage of 1.5 V. The cyclic voltammograms (CVs, *v* = 2 mV s^−1^) and galvanostatic charge/discharge curves (0.1 A g^−1^) before and after the cycling tests were compared. 

## 3. Results and Discussion 

### 3.1. Hybrid Cell 1 with ACC_pristine_(+)/KAC(-) and 1 mol L^−1^ Sodium Iodide 

**Figure 1:**
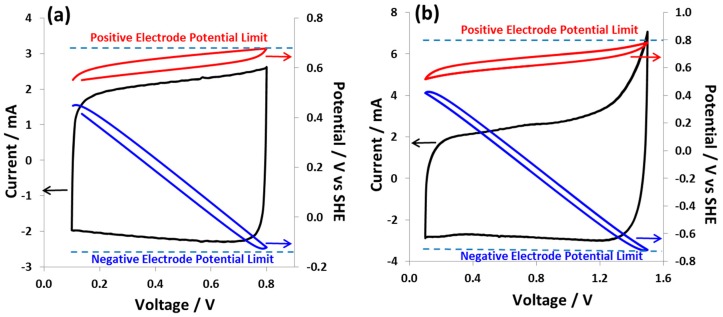
Cyclic voltammograms at *v* = 2 mV s^−1^ and voltage (**a**) 0.1 V to 0.8 V and (**b**) 0.1 V to 1.5 V of carbon/carbon hybrid cell 1. The upper red curve and lower blue curve demonstrate respectively the potential excursion of a positive and negative electrode during cyclic voltammetry.

The stability limit of hybrid cell 1 was determined by polarizing from 0.8 V to 1.5 V with a gradual increase of the maximum voltage from cycle to cycle. Cyclic voltammograms were recorded at a scan rate of 2 mV s^−1^ during which the potential of the individual electrodes was monitored versus the reference electrode. Figure S3 shows the CVs starting from 0.1 V up to 0.8 V and 1.5 V, where an increase of the current at the terminal voltage can be observed for each scan, reaching a maximum at 1.5 V. At the cell voltages 0.8 V (Figure 1a) and 1.5 V (Figure 1b), the positive electrode operates in a narrow potential range, whereas the potential of the negative electrode varies in a wide range. The maximum potential of the positive electrode at a cell voltage of 1.5 V is around 0.79 V vs. SHE and that of the negative electrode reaches −0.76 V vs. SHE. The cyclic voltammogram with a maximum cell voltage of 0.8 V has a capacitor-like rectangular shape, and with the increase of cell voltage this exhibits a more resistive character indicated by a sharp current increase. It is also worth noting that the redox potential of the positive electrode owing to iodide/iodine couple is very close to the cell equilibrium potential, which is the reason for the near-constant potential of the positive electrode for the entire cell voltage up to 1.5 V. Nevertheless, at the maximum voltage of 1.5 V, some contributions from other faradaic processes can be observed, where the potential of the negative electrode reaches down to −0.79 V vs. SHE. At this potential of the negative electrode, reactions such as the reduction of water resulting in hydrogen and hydroxyl ion production may start.

**Figure 2:**
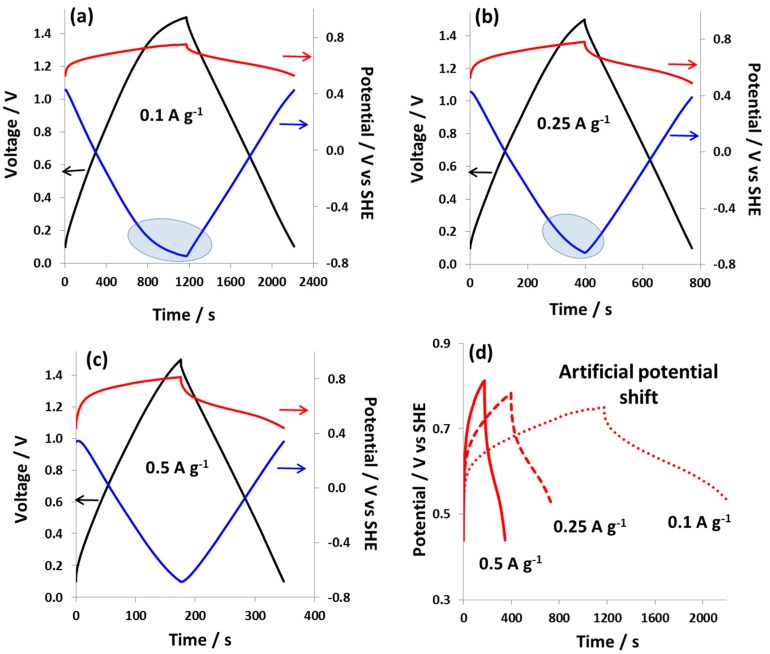
Galvanostatic charge/discharge curves of hybrid cell 1 in a two-electrode setup equipped with s reference electrode with 1 mol L^−1^ NaI electrolyte and specific currents of (**a**) 0.1 A g^−1^, (**b**) 0.25 A g^−1^, (**c**) 0.5 A g^−1^ and (**d**) the charge/discharge curves of the positive battery-like electrode at different specific currents from 0.1 A g^−1^ to 0.5 A g^−1^. Potentials of the positive and negative electrode are plotted in red and blue, respectively.

Figure 2 shows the galvanostatic charge/discharge curves at specific current values of 0.1 A g^−1^ (Figure 2a), 0.25 A g^−1^ (Figure 2b), and 0.5 A g^−1^ (Figure 2c). At a low specific current of 0.1 A g^−1^ and a cell voltage between 0.1–1.5 V, the positive electrode operates in a narrow potential window of 0.21 V (from 0.54 V to 0.75 V vs. SHE) displaying a battery-like charge/discharge behavior. By contrast, the negative electrode works in a large potential window of 1.14 V (from 0.4 V to −0.74 V vs. SHE). However, the hybrid cell at this low current of 0.1 A g^−1^ displays a poor energy efficiency of 68% which is mainly due to the plateau in the charging curve of negative electrode indicating slow kinetics of faradaic processes such as sluggish hydrogen evolution in the presence of iodide species. When the charging current is increased to 0.25 A g^−1^ (Figure 2b), the positive electrode shows a slightly enlarged potential window of 0.29 V, while the potential window of the negative electrode is shifted in a positive direction, improving the hybrid cell energy efficiency to 79%. At an even higher current of 0.5 A g^−1^ (Figure 2c), the charge/discharge curve for the negative electrode is nearly symmetric with a high energy efficiency of 82% for the hybrid cell. The enhanced energy efficiency at high specific currents is the result of smaller losses from parasitic faradaic processes at the negative electrode. Nevertheless, important ohmic loss at the positive electrode with increasing specific current also contributes to the energy efficiency values of the hybrid cell. Figure 2d shows the charge/discharge of the positive battery-like electrode with increasing specific current (from 0.1 A g^−1^ to 0.5 A g^−1^). Clearly, the potential window of the positive electrode is modified at a high current, and consequently it works in a slightly larger potential range indicating an increased contribution from the EDL capacitance. Such an artificial potential shift is controlled by the applied current resulting in decreased contribution (with increasing specific current) of the redox couple to the total capacity of the positive electrode. Galvanostatic charge/discharge curves at specific current of 0.1 A g^−1^ are also shown in Figure S4. Here, the maximum cell voltage was varied up to 1.4 V and a gradual increase of faradaic contributions with increasing cell voltage and increasingly negative potentials at the negative electrode can be observed. 

**Figure 3:**
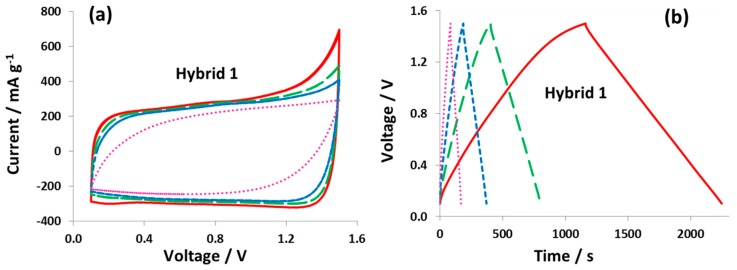
Two-electrode cell performance of hybrid cell 1 with 1 mol L^−1^ NaI electrolyte: (**a**) CVs at 2 mV s^−1^ (full line), 5 mV s^−1^ (big dashed line), 10 mV s^−1^ (small dashed line), and 50 mV s^−1^ (dotted line) and (**b**) GCPL curves at 0.1 A g^−1^ (full line), 0.25 A g^−1^ (big dashed line), 0.5 A g^−1^ (small dashed line), and 1.0 A g^−1^ (dotted line).

Figure 3a presents the cyclic voltammograms of hybrid cell 1 with 1 mol L^−1^ NaI at various scan rates (2 mVs^−1^, 5 mV s^−1^, 10 mV s^−1^, and 50 mV s^−1^), and the capacitance values at each scan rate are given in Table 1. Figure S5 also depicts the CVs up to 1.0 V and 1.5 V at various scan rates. Hybrid cell 1 keeps a high capacitance of 35 F g^−1^ at a scan rate of 50 mV s^−1^. The galvanostatic charge/discharge curves at various specific currents are demonstrated in Figure 3b. The capacitance value for hybrid cell 1 at 0.1 A g^−1^ is 74 F g^−1^ which decreases to 54 F g^−1^ at a specific current of 1.0 A g^−1^ (Table 2). Both at a high scan rate and high current, hybrid cell 1 displays good charge/discharge characteristics. More importantly, the performance of hybrid cell 1 can be recovered when going back to a low scan rate or low specific currents. The energy efficiency calculated from both the cyclic voltammograms and galvanostatic charge/discharge curves is low at a low scan rate and specific current. Once reaching a certain scan rate or specific current, the energy efficiency values remain nearly constant indicating that the redox faradaic processes are dominant at a low sweep rate, consuming more energy during the charging process. The faradaic contributions partly disappear as soon as hybrid cell 1 is cycled at high rates.

### 3.2. Nanostructuration of Battery-Like Electrode Owing to Carbon/Iodide Interface

**Figure 4:**
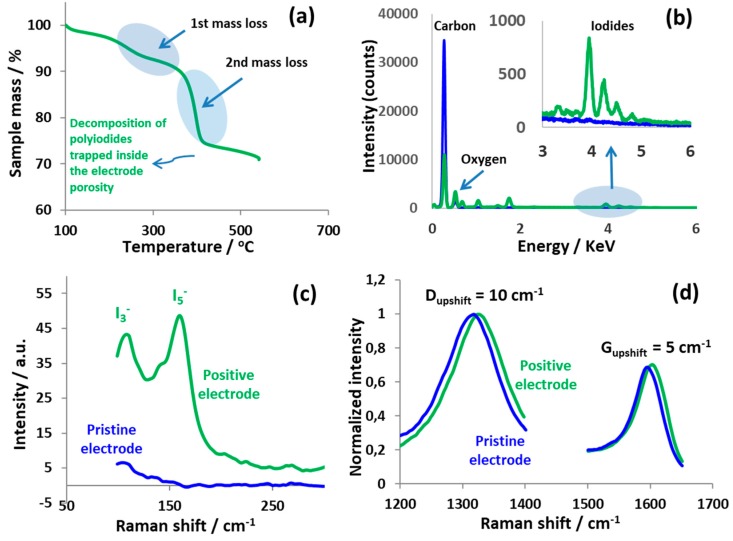
Various analyses on positively polarized ACC electrode (green line) in a hybrid cell using 1 mol L^−1^ NaI up to 1.5 V. (**a**) Thermogravimetric analysis at heating rate of 10 °C/min; (**b**) energy dispersive X-ray analysis compared with pristine material (blue line); (**c**) raman spectra at low wavenumber; and (**d**) D- and G-band region.

After its electrochemical characterization, hybrid cell 1 with 1 mol L^−1^ NaI was disassembled and the positive carbon electrode was analyzed by thermogravimetry (TG) from 100 °C to 550 °C and Raman spectroscopy. Figure 4a shows the TG curve of the positive battery-like electrode, where the mass loss is observed in two steps at a wide temperature range from 190 °C to 425 °C which is due to the decomposition of immobilized polyiodides. The first low mass loss of about 6% from 190 °C to 310 °C is probably due to the iodides present in the mesoporous structure which are decomposed at relatively low temperature. The second mass loss of 17% from 310 °C to 425 °C is due to the polyiodides immobilized inside the micropores of the positive carbon electrode. It has been shown previously that the decomposition temperature of polyiodides trapped in the pores of activated carbon electrodes is in the range of 200 °C to 320 °C [22]. A slightly higher decomposition temperature in the present study might be due to the different polyiodide accommodation sites in the meso-/micropore structure of the carbon material. Nevertheless, a decomposition temperature significantly higher than the usual sublimation temperature of iodine (ca. 80 °C) indicates the trapping of iodide species deep into the micro- and mesopores where it is difficult to be fully extracted. The presence of iodides on the positively polarized ACC electrode in 1 mol L^−1^ NaI is also confirmed by the EDS analysis as demonstrated in Figure 4b. The elemental distribution maps in Figure S6 show that iodine is equally distributed throughout the matrix of the positive carbon electrode. Further, Figure 4c shows the Raman spectra of a pristine carbon electrode and the one positively polarized in hybrid cell 1 with 1 mol L^−1^ NaI. The presence of I_3_^−^ and I_5_^−^ is confirmed by the bands at wavenumbers 110 cm^−1^ and 160 cm^−1^, respectively. However, the Raman spectra of the negative electrode did not show any signal of polyiodides. This is a result of the alkaline conditions developed after the electrochemical reduction of water producing a large excess of hydroxyl ions, which do not favor the formation of polyiodides [50].

The presence of polyiodides inside the pores induces changes in the carbon lattice, which are indicated by the shifts of the D- and G-band as shown in Figure 4d. D- and G-bands are upshifted by 10 cm^−1^ and 5 cm^−1^, respectively, which suggests an extensive charge transfer between the carbon host electrode and the encapsulated polyiodides [35,36,37]. Similar findings have been reported earlier, where a carbon-based electrode was polarized in a bi-functional aqueous electrolyte (Li_2_SO_4_+KI) [23]. 

### 3.3. Design and Performance of Hybrid Cell 2 with ACC_iodide_(+)/KAC (-)Electrodes and 1 mol L^−1^ Sodium Nitrate Electrolyte

**Figure 5:**
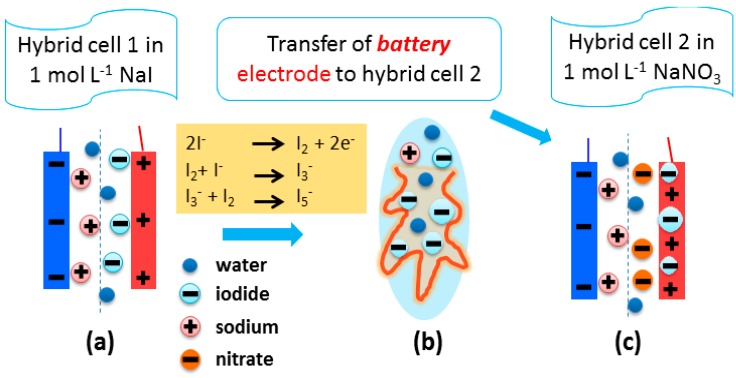
Scheme for (**a**) hybrid cell 1 using 1 mol L^−1^ NaI, and (**b**) battery-like positive electrode is extracted and transferred to (**c**) hybrid cell 2 (using 1 mol L^−1^ NaNO_3_) while keeping the negative electrode from the KOH-activated carbon material.

After confirmation by TG and Raman spectroscopy that polyiodides are immobilized inside the pores of the positive battery-like electrode in hybrid cell 1, a new cell further called *hybrid cell 2* was designed. For this purpose, the positive battery-like electrode from hybrid cell 1 was transferred to the new hybrid cell 2 with 1 mol L^−1^ NaNO_3_ as the electrolyte. Figure 5 shows the schematic of this assembly, where the positive carbon electrode from hybrid cell 1 is transferred after polarization to construct a new hybrid cell 2. Clearly, the addition of redox active species has been avoided in order to prevent the shuttling of iodides to the negative electrode and to achieve stable hybrid cell performance.

**Figure 6:**
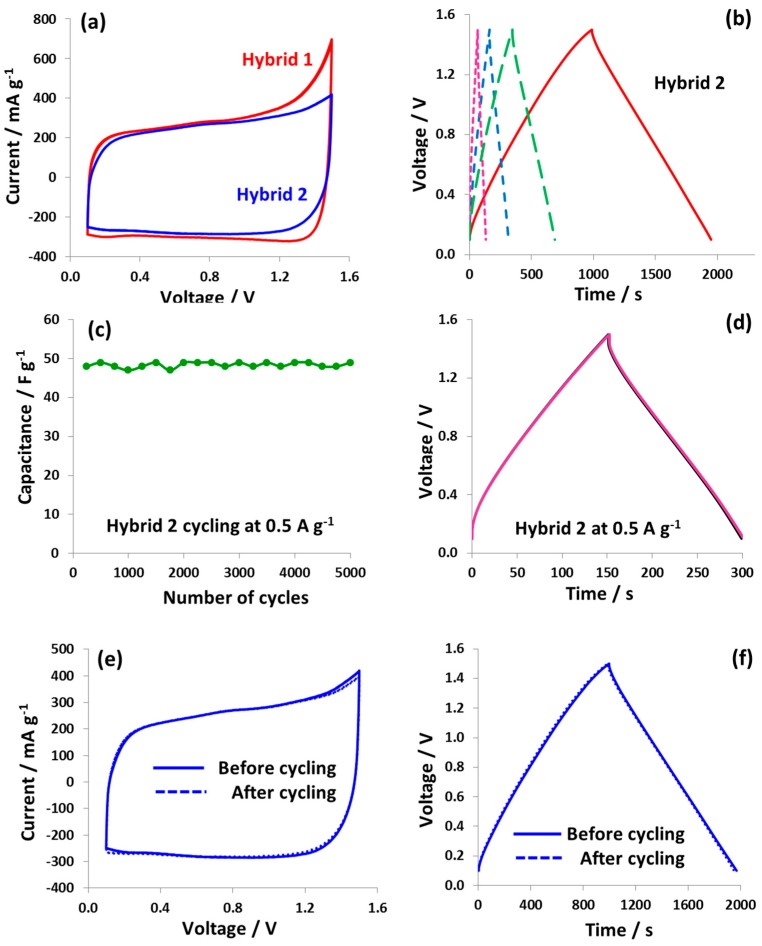
(**a**) Two-electrode cell cyclic voltammograms of hybrid cell 1 and hybrid cell 2; (**b**) galvanostaitic charge/discharge curves of hybrid cell 2 in 1 mol L^−1^ NaNO_3_ at various specific currents of 0.1 A g^−1^ (full line), 0.25 A g^−1^ (big dashed line), 0.5 A g^−1^ (small dashed line), and 1.0 A g^−1^ (dotted line); (**c**) capacitance evolution of hybrid cell 2 for each 250th galvanostatic charge/discharge cycle at 0.5 A g^−1^ over a period of 5000 cycles. (**d**) Comparison of curves after each 1000th galvanostatic charge/discharge cycle; two-electrode cell performance of hybrid cell 2 in 1 mol L^−1^ NaNO_3_ with (**e**) CVs at 2 mV s^−1^ and (**f**) galvanostatic charge/discharge at 0.1 A g^−1^ up to 1.5 V before (full line) and after (dashed line) galvanostatic cycling.

Figure 6a shows the cyclic voltammograms of hybrid cell 1 and 2. In addition, the CVs and galvanostatic charge/discharge curves (at 1.0 V and 1.5 V) for hybrid cell 2 at *v* = 2 mV s^−1^ and a specific current of 0.1 A g^−1^, respectively, are presented in Figure S7. It can be seen that the cyclic voltammogram of hybrid cell 2 is more rectangular and the current rise at the terminal voltage of 1.5 V is far less pronounced compared to that of hybrid cell 1. This improved cyclic voltammetric behavior of hybrid cell 2 suggests an adjustment of the potential range of positive and negative electrodes. Figure 6b depicts the galvanostatic charge/discharge curves at specific currents of 0.1 A g^−1^, 0.25 A g^−1^, 0.5 A g^−1^, and 1.0 A g^−1^. The capacitance decreases from 68 F g^−1^ at 0.1 A g^−1^ to 49 F g^−1^ at 1.0 A g^−1^, which still is quite a high value for such a high specific current. The galvanostatic charge/discharge curves for hybrid cell 1 and hybrid cell 2 at various specific currents are presented in Figure S8. The energy efficiency of hybrid cell 2 has dramatically improved to 84% compared to only 68% for hybrid cell 1 at 0.1 A g^−1^. The cyclability tests at 0.5 A g^−1^ for 5000 galvanostatic charge/discharge cycles demonstrate stable capacitance evolution (Figure 6c) whereas the galvanostatic charge/discharge curves extracted after each 1000 cycles are all superimposed (Figure 6d). Moreover, the cyclic voltammograms (2 mVs^−1^) and galvanostatic charge/discharge (0.1 A g^−1^) before and after 5000 cycles also remain unchanged as shown in Figure 6e,f, confirming the good state of health of hybrid cell 2. Table S2 shows the comparison of capacitance evolution during galvanostatic charge/discharge cycling reported previously in the literature and the present newly designed hybrid cell. Clearly, hybrid cell 2 does not show any capacitance decay while other systems have demonstrated capacitance loss in a wide range from 8% to 35% (as a result of iodine shuttling) during a given cycling period. 

**Figure 7:**
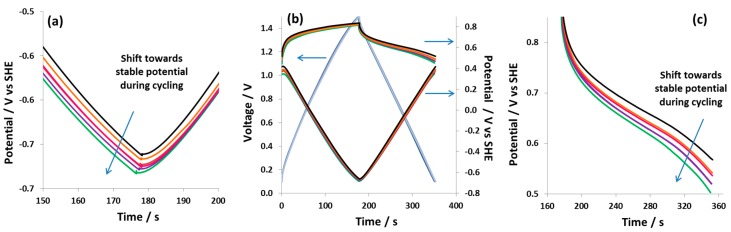
Galvanostatic charge/discharge curves (0.5 A g^−1^) of hybrid cell 2 obtained in a two-electrode cell equipped with a reference electrode after every 1000th galvanostatic charge/discharge cycle (**a**) negative EDL electrode, (**b**) full cell, and (**c**) positive battery-like electrode.

Figure 7a–c shows the galvanostatic charge/discharge curves at 0.5 A g^−1^ for hybrid cell 2, which was equipped with a reference electrode. Each galvanostatic charge/discharge curve was obtained after 1000 cycles in order to observe the changes in electrochemical characteristics during cycling. During 5000 cycles, both the positive battery-like electrode and the negative EDL electrode show a small shift of potential in a negative direction. This potential shift could be due to the dissolving of iodide species present at the electrode surface into the electrolyte. It is worth noting that despite the potential shift observed in Figure 7a,c, the capacitance values of hybrid cell 2 remain nearly constant at 48 F g^−1^ during 5000 galvanostatic charge/discharge cycles. The constant evolution of capacitance and the consistent charge/discharge characteristics indicate a stable electrochemical behavior of the battery-like positive electrode and also of hybrid cell 2. 

**Figure 8:**
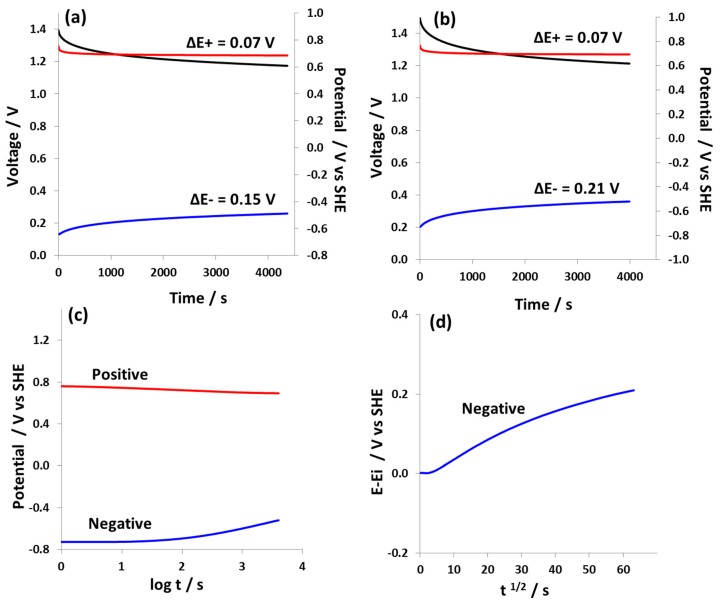
Self-discharge of hybrid cell 2 equipped with a reference electrode at cell voltage of (**a**) 1.4 V and (**b**) 1.5 V; (**c**) potential profiles of positive and negative electrodes versus log t and (**d**) and E-Ei versus t^1/2^ for the negative EDL electrode.

Figure 8 shows the self-discharge behavior of hybrid cell 2 charged to 1.4 V and 1.5 V. The positive battery-like electrode displays a very low potential decay of 0.07 V at both 1.4 V (Figure 8a) and 1.5 V (Figure 8b). The negative electrode, on the other hand, which operates down to rather negative potentials, shows high potential decay at both 1.4 V and 1.5 V cell voltage. This strong potential decay of the negative electrode can be explained by the contribution of redox processes related to the electrochemical reduction of water at large negative potentials. These redox processes result in the generation of hydroxyl anions, which causes a local shift of pH in the pores of the negative electrode [51]. Since the concentration of the NaNO_3_ electrolyte is 1 mol L^−1^, which is not very high, the local concentration of OH^-^ generated due to the reduction of water is immediately shifted due to the dilution effect and a consequent shift of local pH to a neutral value occurs, which results in high self-discharge at the negative electrode, as explained elsewhere [52,53]. Overall, the self-discharge of hybrid cell 2 in 1 mol L^−1^ NaNO_3_ is determined by the negative electrode. Figure 8c shows the comparison of potential profiles of positive and negative electrodes of hybrid cell 2 versus log t, which suggests the high impact of activation-controlled mechanisms on the discharge processes at the negative electrode, which is probably related with the electrochemical reduction of water [54,55]. This is also confirmed by Figure 8d, where the E-E_i_ versus t^1/2^ graph shows non-linear behavior indicating that discharge processes at the negative electrode are less controlled by the diffusion of impurities/redox species [55]. 

## 4. Conclusions

The immobilization of polyiodides inside nanoporous carbon is enhanced by the strong charge transfer between carbon host and iodides forming a C-I complex which works as a solid redox electrode. The high capacity of this electrode was harnessed by coupling with a carbon electrode of high EDL capacitance in a cell where the electrolyte does not contain any redox-active species. The new hybrid cell displayed symmetric charge/discharge curves and nearly rectangular cyclic voltammograms with excellent cyclability up to 1.5 V at a high specific current. The concept of using iodated carbon for designing hybrid cells can be further extended by changing the porous structure and surface chemistry of the carbon material. By this way, the extent of charge transfer between carbon and iodide species could be improved. Due to the numerous advantages of aqueous over organic electrolyte media, hybrid devices with a concentrated electrolyte can also be assembled to reduce the effects of local pH shift under dilution effect as described in Ref. [52], and consequently, further reduce the self-discharge of the hybrid cell.

## Figures and Tables

**Table 1:** Capacitance and energy efficiency values calculated from cyclic voltammograms at each scan rate for hybrid cell 1.

	2 mV s^−1^	5 mV s^−1^	10 mV s^−1^	50 mV s^−1^
**Capacitance (F g^−1^)**	72	65	59	35
**Energy Efficiency (%)**	92	97	97	98

**Table 2:** Capacitance and energy efficiency values calculated from galvanostatic charge/discharge curves at each specific current for hybrid cell 1.

	0.1 A g^−1^	0.25 A g^−1^	0.5 A g^−1^	1.0 A g^−1^
**Capacitance (F g^−1^)**	74	71	63	54
**Energy Efficiency (%)**	68	79	82	82

## Supplementary Materials

The following are available online at https://www.mdpi.com/2079-4991/9/10/1413/s1, Figure S1: pore size distribution of carbons, Table S1: porous data of carbons, Figure S2: SEM image of pristine ACC, Figure S3: CVs of hybrid cell 1, Figure S4: Charge/discharge curves of hybrid cell 1, Figure S5: CVs of hybrid cell 1 at high scan rates, Figure S6: SEM images of positively polarized ACC, Figure S7: CVs and charge/discharge curves of hybrid cell 2, Figure S8: Comparison of hybrid cell 1 and hybrid cell 2 performance.
