# Supplementary material for: Immobilization of Polyiodide Redox Species in Porous Carbon for Battery-Like Electrodes in Eco-Friendly Hybrid Electrochemical Capacitors
Source: Nanomaterials (Basel). 2019 Oct 3;9(10):1413. doi: 10.3390/nano9101413 (PMC6835913; doi:10.3390/nano9101413)
Supplement: Supplementary file 1 [file nanomaterials-09-01413-s001.pdf]

Article

Immobilization of Polyiodide Redox Species in Porous Carbon for Battery-Like Electrodes in Eco-Friendly Hybrid Electrochemical Capacitors

Q. Abbas [1,*,†], H. Fitzek [2], H. Schroettner[2,3], S. Dsoke [4] and B. Gollas [1]

[1] Institute for Chemistry and Technology of Materials, Graz University of Technology, 8010 Graz, Austria; Bernhard.Gollas@tugraz.at

[2] Graz Centre for Electron Microscopy, Steyrergasse 17, 8010 Graz, Austria; harald.fitzek@felmi-zfe.at (H.F.); hartmuth.schrottner@felmi-zfe.at (H.S.)

[3] Institute of Electron Microscopy and Nanoanalysis, NAWI Graz, Graz University of Technology, Steyrergasse 17, 8010 Graz, Austria

[4] Institute for Applied Materials (IAM), Karlsruhe Institute of Technology (KIT), Hermann-von-Helmholtz-Platz 1, 76344 Eggenstein-Leopoldshafen, Germany; sonia.dsoke@kit.edu

* Correspondence: qamar.abbas@tugraz.at

† The author is currently on leave from Poznan University of Technology, Poland

Table 1. Porous data of KOH activated carbon (KAC) and activated carbon cloth (ACC).

Activated carbon	Surface area (m^2 g^{-1})	Micropore volume (cm^3 g^{-1})	Mesopore volume (cm^3 g^{-1})
ACC	2320	0.84	0.06
KAC	2300	0.88	0.02

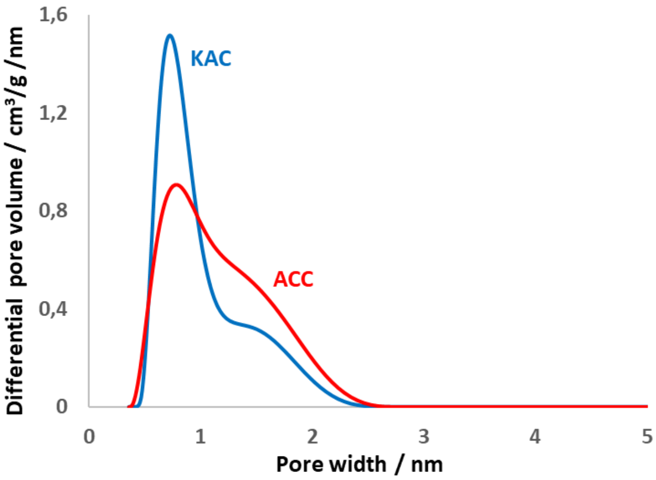

Figure 1. Pore size distribution of KAC and activated carbon cloth (ACC).

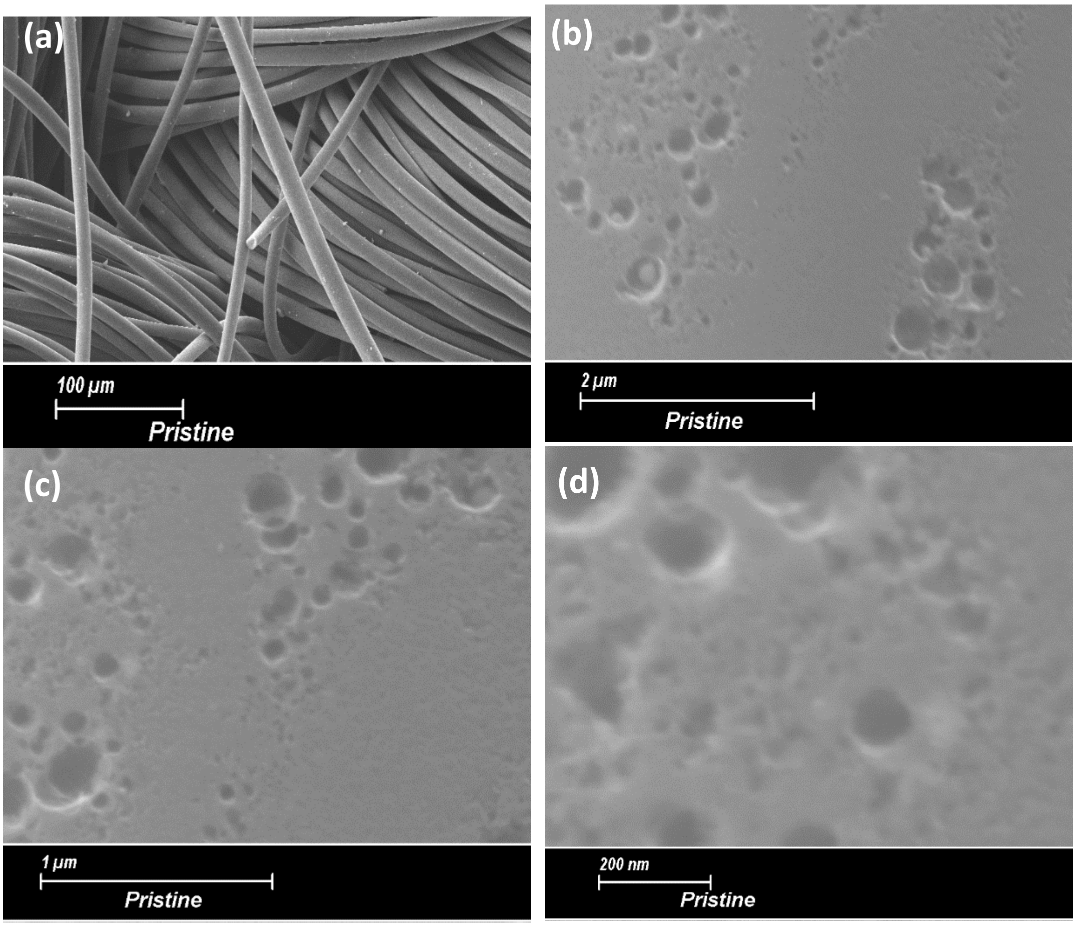

Figure 2. Scanning electron micrographs of pristine activated carbon cloth (ACC 507-20).

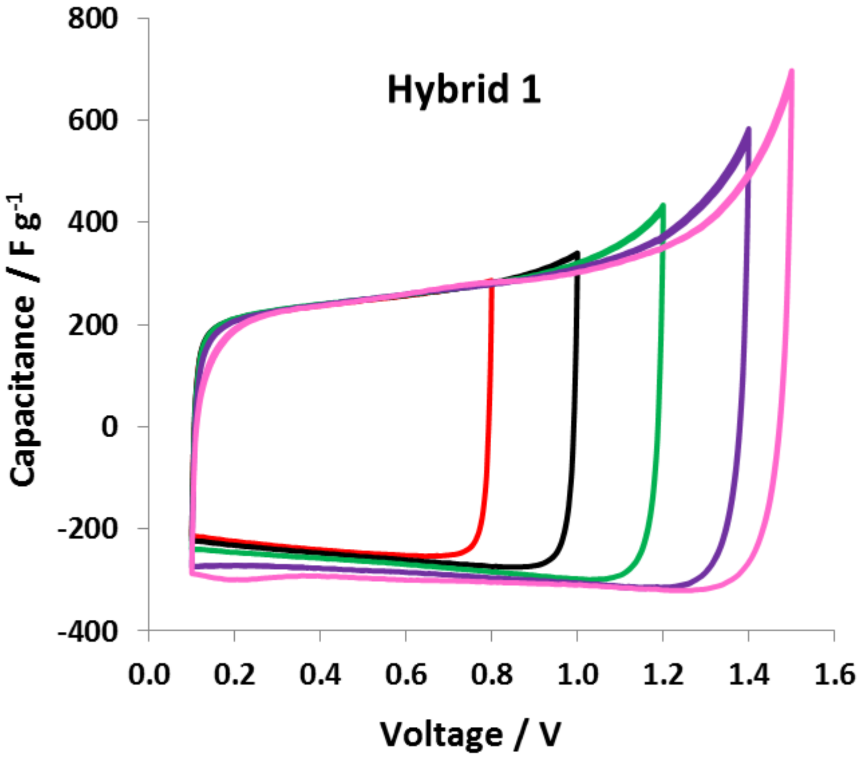

Figure 3. Cyclic voltammograms of hybrid cell 1 in 1 mol L^{-1} NaI at 2 mV s^{-1} with gradual voltage increase from 0.1 V up to 1.5 V.

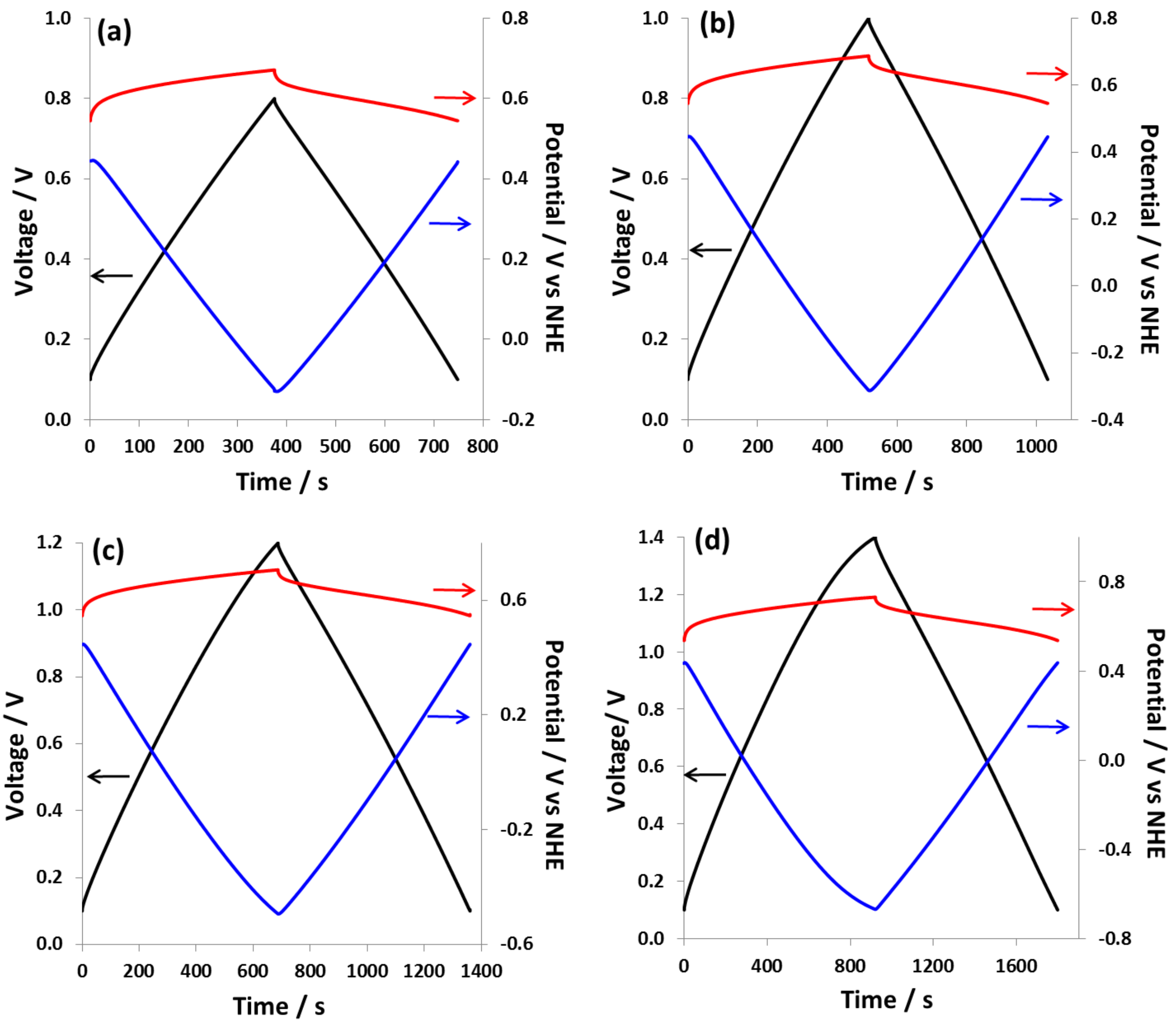

Figure 4. Galvanostatic charge/discharge curves of hybrid cell 1 in two electrode cell equipped with reference electrode in 1 mol L^{-1} NaI as electrolyte at specific current of 0.1 A g^{-1} up to cell voltage (a) 0.8 V, (b) 1.0 V, (c) 1.2 V and (d) 1.4 V.

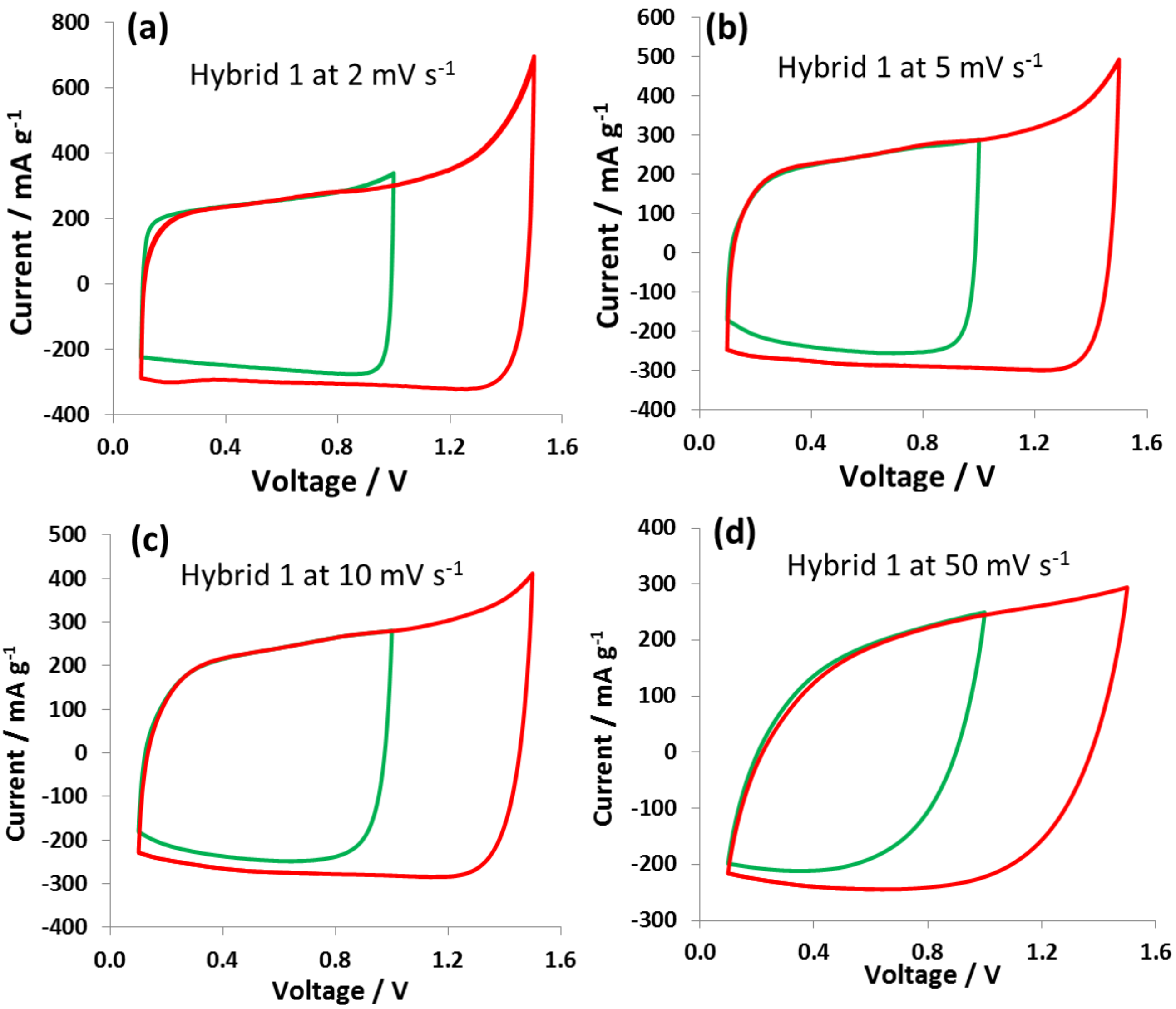

Figure 5. Two electrode cell performance of hybrid cell 1 in 1 mol L^{-1} NaI with CVs up to 1.0 V and 1.5 V using scan rate of (a) 2 mV s^{-1}, (b) 5 mV s^{-1}, (c) 10 mV s^{-1} and (d) 50 mV s^{-1}.

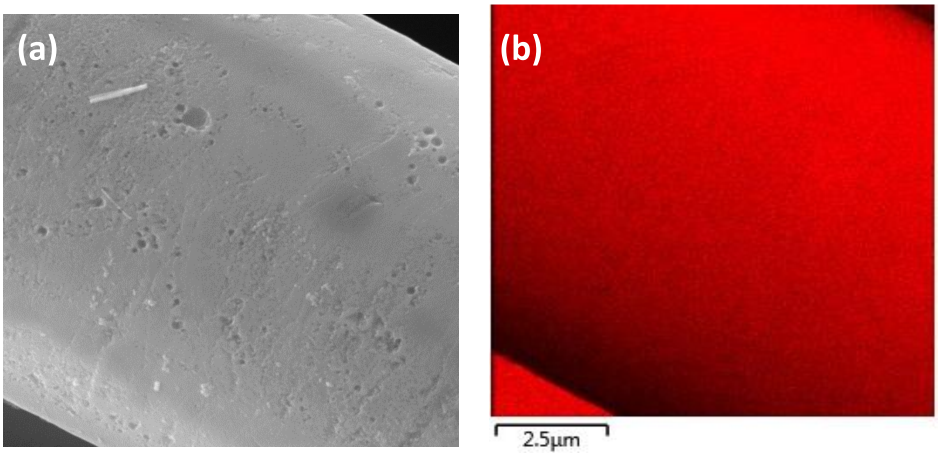

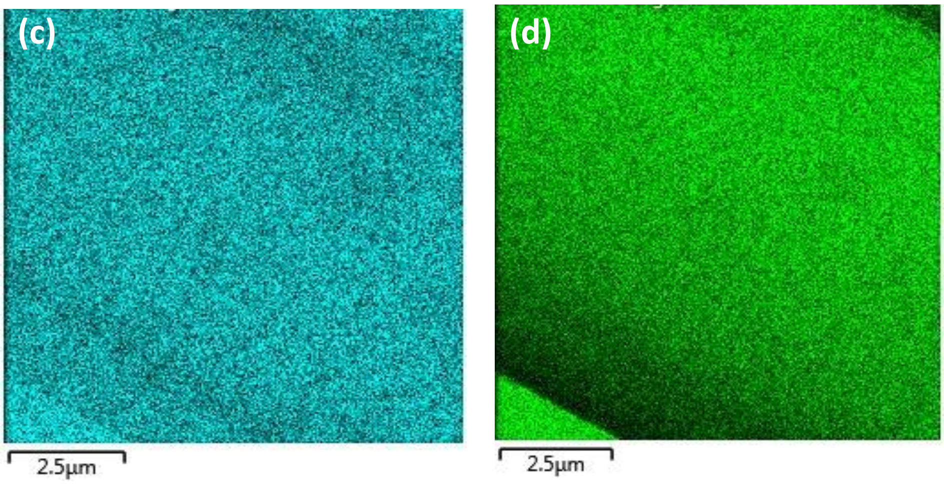

Figure 6. (a) Scanning electron micrograph of positive ACC electrode with corresponding surface mapping of (b) carbon, (c) iodine and (d) sodium.

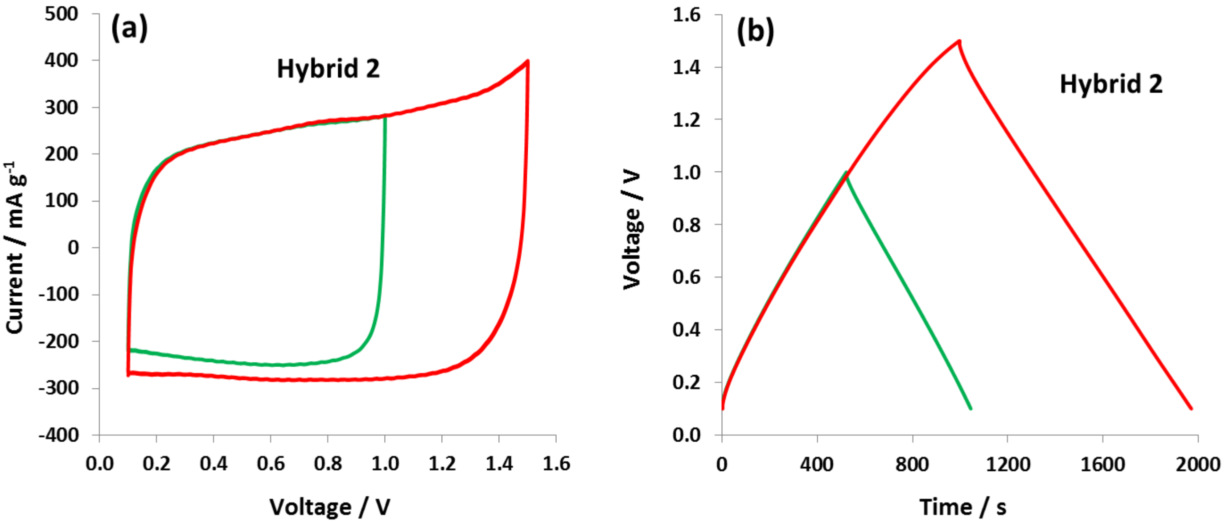

Figure 7. Two electrode cell performance of hybrid cell 2 in 1 mol L^{-1} $NaNO_3$ with (a) CVs and (b) galvanostatic charge/discharge up to 1.0 V and 1.5 V using scan rate of 2 mV s^{-1} and specific current of 0.1 A g^{-1} respectively.

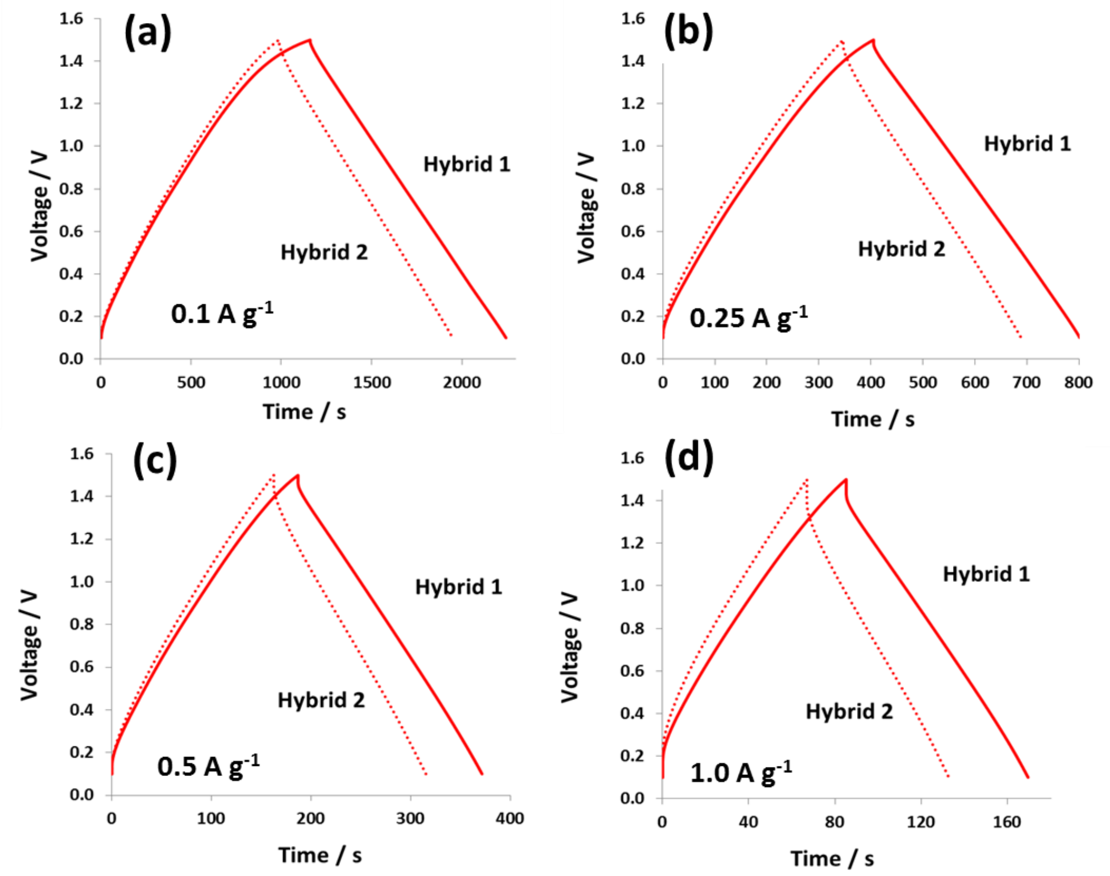

Figure 8. Comparison of two electrode cell performance of hybrid cell 1 in 1 mol L^{-1} NaI and hybrid cell 2 in 1 mol L^{-1} $NaNO_3$ with galvanostatic charge/discharge up to 1.5 V using specific current (a) 0.1 A g^{-1}, (b) 0.25 A g^{-1}, (c) 0.5 A g^{-1} and (d) 1.0 A g^{-1}

Table 2. Performance comparison during 5000 galvanostatic charge/discharge cycles for various hybrid cells using iodides as redox active specie in aqueous electrolyte.

Electrolyte.	Cell voltage	Number of galvanostatic charge/discharge cycles	Percentage of capacitance loss	Ref.
$KI+Na_2MoO_4$	1.0 V	5000	35.4%	1
LiTFSI+KI	1.8 V	10000	21%	2
Li_2SO_4+KI	1.6 V	10000	9%	3
$MnSO_4$+KI	1.5 V	10000	8%	4
$ChNO_3$+ChI	1.5 V	20000	8%	5
$NaNO_3$	1.5 V	5000	No capacitance decay	This work

References:

1. D. Xu, W. Hu, X. Na Sun, P. Cui, X. Ying Chen, *J. Power Sources* 341 (2017) 448-456.
2. D. Reber, R-S. Kühnel, C. Battaglia, *Sustainable Energy Fuels*, 1 (2017) 2155-2161.
3. Q. Abbas, P. Babuchowska, E. Frackowiak, F. Béguin, J. *Power Sources* 326 (2016) 652-659.
4. P. Przygocki, Q. Abbas, P. Babuchowska, F. Béguin, *Carbon* 125 (2017) 391-400.

5. P. Przygocki, Q. Abbas, B. Gorska, F. Béguin, *J. Power Sources* 427 (2019) 283-292.
